# ATP dependence of decision-making capacity in a fine-grained model of gene regulatory networks

**DOI:** 10.1098/rsif.2024.0246

**Published:** 2024-06-19

**Authors:** Rajneesh Kumar, Iain G. Johnston

**Affiliations:** ^1^ Department of Mathematics, University of Bergen, Bergen, Norway; ^2^ Computational Biology Unit, University of Bergen, Bergen, Norway

**Keywords:** cellular decision-making, gene regulatory networks, systems biology

## Abstract

Cellular decision-making is fundamental to life, from developmental biology to environmental responses and antimicrobial resistance. Many regulatory processes that drive cellular decisions rely on gene expression, which requires energy in the form of ATP. As even genetically identical cells can have dramatically different ATP levels, bioenergetic status can be an important source of variability in cellular decision-making. Existing studies have investigated this energy dependence but often use coarse-grained modelling approaches (which are not always readily connected to the underlying molecular processes of gene regulation). Here, we use a fine-grained mathematical model of gene expression in a two-gene decision-making regulatory network to explore cellular decision-making capacity as energy availability varies. We simulate both a deterministic model, to explore the emergence of different cell fate attractors as ATP levels vary, and a stochastic case to explore how ATP influences the noisy dynamics of stochastic cell decision-making. Higher energy levels typically support increased decision-making capacity (higher numbers of, and more separated, cell states that can be selected), and the fine-grained modelling reveals some differences in behaviour from previous coarse-grained modelling approaches.

## 1. Introduction

The supply of energy is fundamental to the out-of-equilibrium processes that maintain life, from physical motion and metabolism to gene expression and information processing [1–3]. ATP molecules serve as primary energy carriers in cells: many essential biological processes, including gene expression, metabolism and transport, are regulated by ATP, which serves as a universal cofactor for cellular energy. The expression of genes, involving ATP-consuming transcription and translation, fundamentally determines a cell’s behaviour and its response to given conditions. The substantial ATP differences that exist across cells in a wide variety of organisms [4–7] can, therefore, give rise to substantial differences in gene expression and cell behaviour.

Gene regulatory networks (GRNs), where sets of genes influence each others’ expression, underlie a host of cell decision-making and information-processing mechanisms [8–12]. A GRN may support a range of different expression profiles, each corresponding to a different cell state or cell fate. The response of GRNs to different environmental conditions, and the consequent changes in cell behaviour supported by GRN dynamics, give a cell ‘decision-making’ capacity—that is, the ability to ‘decide’ on a particular state of gene expression [13,14].

Changes in gene expression profiles, and thus in cell behaviour, may be driven by random chance as well as external stimuli. The discrete nature of molecular processes and the inherent randomness in reaction occurrences are commonly referred to as ‘intrinsic noise’ at the molecular level of gene expression, where transcription and translation are carried out in environments with only a relatively small number of copies [15,16]. When molecular substances like DNA, mRNA and regulatory proteins become scarce, there is intrinsic noise in biochemical processes [8,12,17] that causes variability in their activity. Such variation may influence the dynamics of GRNs and cellular decision-making processes, causing unpredictability and uncertainty in the cellular response [18–22]. This influence of noise may cause qualitative changes in behaviour, for example, supporting multiple stable states where a deterministic picture only supports one—a phenomenon called noise-induced bi- or multi-stability [23–25]. Intrinsic noise is now generally acknowledged to play important roles in GRN behaviour [20,22,26]. It improves a single phenotype’s capacity to adapt to shifting environmental conditions [18,21] and can fundamentally shape cell decision-making [14,27,28].

A classic illustration of cellular decision-making in response to environmental stimuli is the Lac operon, where a bacterial cell carefully regulates lactose uptake and metabolism by regulating the operon’s expression in response to external factors [29]. When lactose is in limited supply, the operon remains suppressed to conserve energy. However, lactose acts as an inducer when it is present, triggering the operon to be activated to promote lactose metabolism. With the support of this regulatory mechanism, the cell possesses the capability to efficiently adapt its processes of metabolism to changing environmental conditions.

Another example of bacterial decision-making reflects the influence of randomness. Persisters, stochastic variants in microbial populations, are characterized by their dormancy and high antibiotic tolerance. A drop in cytoplasmic ATP levels, along with stochastic changes in cell behaviour, is linked to the formation of persister cells across different species [30–32], contributing to their antibiotic tolerance and dormancy (although the universality of this link is debated [33]).

The role of ATP in gene expression [1–3,34,35], and the fact that substantial cell-to-cell diversity can exist in ATP concentrations in different systems [4–7] means that ATP variability can lead to cell-to-cell variability in GRN dynamics and behaviour. This mirrors the ATP dependence of other biomolecular pathways [36–38]—for example, the detailed role of ATP in shaping the dynamics of signalling cascades has recently been illustrated using quantitative modelling grounded in systems biology and thermodynamics [39]. Theoretical studies have shown that simplified GRN models [40], using coarse-grained descriptions of genes and gene expression processes, exhibit strong energy-dependent diversity in decision-making capacity [41–43]. In such models of simple mutually repressing GRN motifs, increased ATP concentrations led to more attractor states (that is, distinct stable states of gene expression to which other states evolve over time) and, hence, to more decision-making capacity in cells. Lower ATP concentrations reduced the attractors supported to the point where only a single mode of behaviour was permitted, compromising decision-making ability. Assuming that ATP concentration scales the rate of gene expression processes [34], this behaviour is compatible with classic studies on the attractor structure of model GRNs [44,45], which typically find that increased rates of gene expression support more, and more stable, dynamic attractors.

Although informative, many of these studies used coarse-grained models of gene expression [40,46], neglecting differences between RNA and protein (using a single, combined ‘gene’ variable to track expression) and a heuristic Hill function model for gene interactions. Further, the dynamics of gene expression are fundamentally stochastic, but the investigation of ATP influence on stochastic models of GRN dynamics is limited. The question therefore remains open: do more realistic models of GRN behaviour also show energy dependence on decision-making?

To address this, we implemented deterministic and stochastic versions of a GRN model that explicitly tracks DNA, RNA and protein concentrations, where dimerized proteins act to control gene expression in a mutually antagonistic motif, from Kim & Gelenbe [47] and ultimately deriving from Gardner *et al*. [44], with parameters and reactions all corresponding to empirically measurable observables. We explored conditions under which this model demonstrates decision-making capacity (the appearance of more than one attractor) and how this capacity depends on ATP.

## 2. Methods

### 2.1. Model description

**Figure 1.**
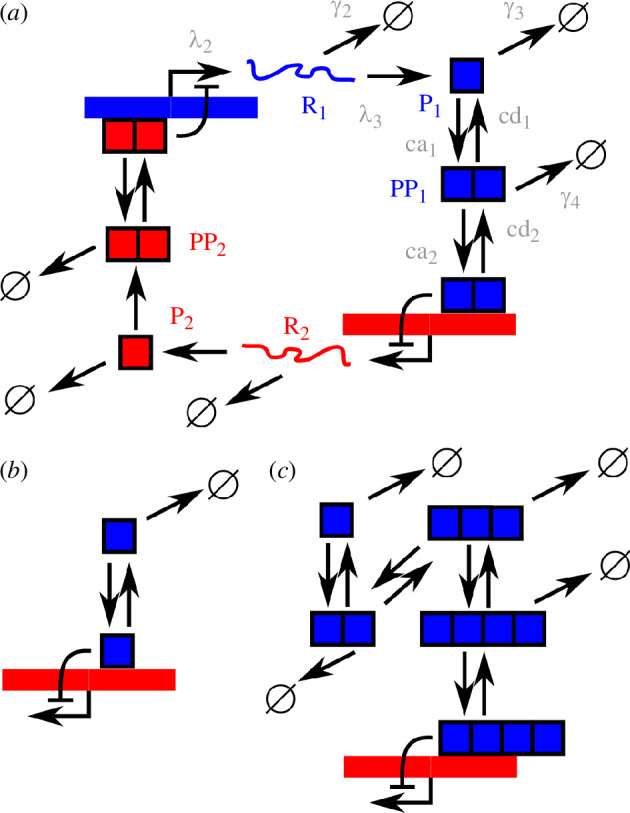
Schematic of the model system. (*a*) Default system, where P_1_ and P_2_ are expressed and their dimers repress each others’ expression. For clarity, rates are only shown on processes involving the first product; they apply symmetrically to the other. *λ*_2_ and *λ*_3_ will be scaled by model ATP concentration to capture ATP dependence of transcription and translation. (*b*) Subset of the simplified system without polymerization, where the monomer acts as the regulatory factor. (*c*) Subset of the more complicated system where oligomerization proceeds up to the homotetramer, which acts as the regulatory factor.

We use the switch model from Kim & Gelenbe [47], following the classical toggle switch model in Gardner *et al*. [44], but remove the delay aspect of translation to simplify the system. The model includes two genes *i* and *j*, the protein products of which dimerize. The dimer of one gene’s product inactivates the promoter of the other (figure 1*a*). In other model variants (figure 1*b*,*c*; table 1), we introduce further (or fewer) polymerization processes, so that the polymer of size *n* is the regulatory factor influencing gene expression, where *n* ranges from 1 (monomer regulation) to 4 (tetramer regulation). We will mainly focus on the well-studied *n *= 2 case.

**Table 1.** Processes in the model.

process	reaction	rates (default parameterization value in s^−1^)
transcription from active promoter	Pro_*i*_ + TF_*i*_→ Pro_*i*_ + TF_*i*_ + R_*i*_	*λ*_2_ (0.0067)
translation	R_*i*_→ R_*i*_ + P_*i*_	*λ*_3_ (0.0474)
dimerization/dedimerization	P_*i*_ +P_*i*_ ↔ PP_*i*_	ca_1_ forward / cd_1_ backward (0.0023 / 0.00023)
dimer binding/unbinding to promoter*	Pro_*i*_ + PP_*j*_ ↔ Pro_*i*_ PP_*j*_	ca_2_ forward / cd_2_ backward (0.1038 / 1.04)
RNA degradation	R_*i*_ → 0	*γ*_2_ (0.00023)
protein degradation	P_*i*_ → 0	*γ*_3_ (0.00077)
dimer degradation	PP_*i*_→ 0	*γ*_4_ (0.00058)
oligomerization model variant processes (only one promoter interaction * is present in any model instance)		
monomer binding/unbinding to promoter*	Pro_*i*_ + P_*j*_ ↔ Pro_*i*_ P_*j*_	ca_2_ / cd_2_ (0.1038 / 1.04)
trimerization/detrimerization	P_*i*_ +PP_*i*_ ↔ PPP_*i*_	ca_1_ / cd_1_ (0.0023 / 0.00023)
trimer binding/unbinding to promoter*	Pro_*i*_ + PPP_*j*_ ↔ Pro_*i*_ PPP_*j*_	ca_2_ / cd_2_ (0.1038 / 1.04)
trimer degradation	PPP_*i*_→ 0	*γ*_4_ (0.00058)
tetramerization/detetramerization	P_*i*_ +PPP_*i*_ ↔ PPPP_*i*_	ca_1_ / cd_1_ (0.0023 / 0.00023)
tetramer binding/unbinding to promoter*	Pro_*i*_ + PPPP_*j*_ ↔ Pro_*i*_ PPPP_*j*_	ca_2_ / cd_2_ (0.1038 / 1.04)
tetramer degradation	PPPP_*i*_→ 0	*γ*_4_ (0.00058)

We use Kim & Gelenbe’s [47] parameterization, itself taken from the biological literature [20,48–51]. We set transcription factor concentrations equal to a unit constant, reflecting a constant constitutive level of expression, and we initialize the system with Pro_*i*_ = 1 (one promoter for each gene), initial protein levels P_1_ = P_1_*, P_2_ = P_2_*, and all other species at zero concentration. We will model ATP dependence by scaling gene expression processes by a constant given by a model ATP value [42,43], capturing the dependence of transcription and translation rates on ATP concentration [34].

### 2.2. Differential equation model

We explored both deterministic and stochastic instances of this system. The state variables in our system are illustrated in figure 1: p_*i*_ is the monomer protein of gene *i* and pp_*i*_ is the dimer of this protein, pro_*i*_ is the promoter for gene *i*, propp_*i*_ is promoter *j* bound by dimer pp_*i*_, and rna_*i*_ is mRNA for gene *i*. *z*_*i*_ is the constant, unit level of an independent transcription factor, reflecting a baseline level of constitutive expression. For the deterministic picture, we used the governing ordinary differential equations to capture a mass action model of the processes in table 1:


(2.1) $$dpro_{1}/dt=cd_{2}.propp_{2}-ca_{2}.pro_{1}.pp_{2},$$

(2.2) $$dpro_{2}/dt=cd_{2}.propp_{1}-ca_{2}.pro_{2}.pp_{1},$$

(2.3) $$dpropp_{2}/dt=-cd_{2}.propp_{2}+ca_{2}.pro_{1}.pp_{2},$$

(2.4) $$dpropp_{1}/dt=-cd_{2}.propp_{1}+ca_{2}.pro_{2}.pp_{1},$$

(2.5) $$drna_{1}/dt=\lambda_{2}.pro_{1}.z_{1}-\gamma_{2}.rna_{1},$$

(2.6) $$drna_{2}/dt=\lambda_{2}.pro_{2}.z_{2}-\gamma_{2}.rna_{2},$$

(2.7) $$dp_{1}/dt=\lambda_{3}.rna_{1}-2.ca_{1}.p_{1}^{2}+2.cd_{1}.pp_{1}-\gamma_{3}.p_{1},$$

(2.8) $$dp_{2}/dt=\lambda_{3}.rna_{2}-2.ca_{1}.p_{2}^{2}+2.cd_{1}.pp_{2}-\gamma_{3}.p_{2},$$

(2.9) $$dpp_{1}/dt=ca_{1}.p_{1}^{2}-cd_{1}.pp_{1}-ca_{2}.pp_{1}.pro_{2}-\gamma_{4}.pp_{1}+cd_{2}.propp_{1},$$

(2.10) $$dpp_{2}/dt=ca_{1}.p_{2}^{2}-cd_{1}.pp_{2}-ca_{2}.pp_{2}.pro_{1}-\gamma_{4}.pp_{2}+cd_{2}.propp_{2},$$

with the following initial conditions:


(2.11) $$pro_{1}=pro_{2}=1,propp_{1}=propp_{2}=0,z_{1}=z_{2}=1,pp_{1}=pp_{2}=0,rna_{1}=rna_{2}=0.$$

We also considered an alternative model where protein dimerization occurs when a monomer is bound to DNA, outlined in electronic supplementary material, table S1.

### 2.3. Numerical implementation

The system exhibited dynamics which made numerical solution somewhat awkward with some solvers. Specifically, the system quickly relaxes to a set of states where future time behaviour is very slow (electronic supplementary material, figure S1). Several solvers misassessed this slow behaviour as stationarity and did not proceed to the true steady-state solution. To avoid such artefacts, we used a hard-coded forward Euler solver with a convergence criterion defined over a time window and confirmed that an LSODA (Livermore solver for ordinary differential equations) solver in R [52] and a hard-coded Runge–Kutta fourth-order solver (electronic supplementary material, figure S2) also successfully negotiated this slow-changing dynamic. For the stochastic simulation, Gillespie’s stochastic simulation algorithm [53] was used to explicitly simulate trajectories from the processes in table 1.

**Figure 2.**
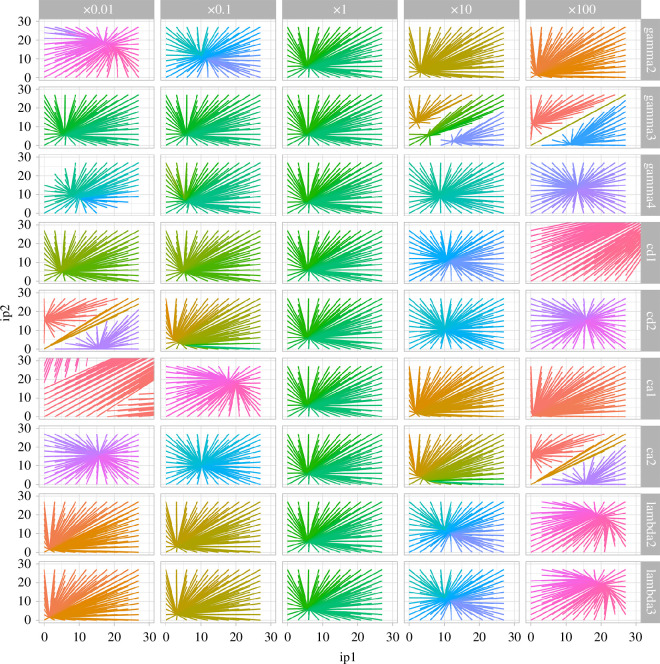
Attractor basin structure with orders-of-magnitude scans through parameters. Each line describes how the given initial condition (ip_1_, ip_2_) evolves to a long-term position in the (p_1_, p_2_) space of protein expression. Rows correspond to different model parameters being varied; columns correspond to the scaling applied to each parameter. Colours are assigned based on the coordinates of the long-term state. Tripartite attractor structure is caused by lower values of transcription factor unbinding rate cd_2_ and higher values of protein degradation rate *γ*_3_ (and, on a different scale, lower values of dimerization rate ca_1_). Missing lines represent situations in which the system failed to converge over the simulated timescale.

The numerical simulations were performed using custom code in C for speed, with some checking using *deSolve* [52] in R. For data handling and visualization, we used R libraries *ggplot2* [54], *reshape* [55] and *ggpubr* [56]. All code is freely available at https://github.com/StochasticBiology/energy-decisions (Zenodo: https://zenodo.org/doi/10.5281/zenodo.11123177).

## 3. Results

### 3.1. Parameter values supporting decision-making capacity in the deterministic model

Within the context of this model, cellular decision-making capacity is defined as the support of distinct cell states defined by protein concentrations (resembling different *in vivo* cellular phenotypes). If the system always converges to a single state, there is no capacity to ‘make a decision’ and adopt a different phenotype according to context. By contrast, if several distinct cellular states are supported by the system, the ability to ‘decide’ between them is supported. States to which a system converges over time are called ‘attractors’, and the set of initial states from which an attractor is eventually reached is called an ‘attractor basin’.

To identify parameterizations of the model that led to non-trivial decision-making capacity, we conducted a stepwise sweep through parameter values over orders of magnitude and looked for those parameterizations that resulted in an attractor basin structure with more than one attractor. Our parameter sweep centred on the biologically derived parameters from [47] and varied each over surrounding orders of magnitude. The support for more than one attractor demonstrates that the system can exhibit different steady states (behaviours) in response to different stimuli or environmental conditions.

During this investigation, we found that the dynamics of the system exhibited rather stiff characteristics: the system rapidly evolves to occupy a region of state space then slowly evolves further within this set of states before finally reaching an attractor. In several instances, the system showed non-monotonic behaviour with time in all state variables (electronic supplementary material, figure S1), with coincident turning points resembling a ‘false convergence’ to a steady state. We tested for convergence over a longer time period to avoid numerical issues with the ODE solvers involved (Methods; electronic supplementary material, figure S2).

The results of this parameter sweep for the default system where dimers are the active regulatory molecules (figure 1*a*) are shown in figure 2. We found in particular that increased protein degradation (*γ*_3_) and decreased TF–DNA dissociation (cd_2_) supported a tripartite system where one central attractor (with equal protein levels) was flanked by two attractors (high pro_1_ and high pro_2_, respectively). In cases where this tripartite structure was not present, the system was typically characterized by relaxation towards a single attractor.

Taking the next step in a combinatorial exploration of the system, we next asked how the attractor structure of the system changed when changes were applied to two parameters simultaneously. For small changes, scaling pairs of parameters by factors of 1/2 or 2, no changes to the single attractor state were observed (electronic supplementary material, figure S3). When larger-scale perturbations were applied, scaling pairs of parameters by factors of 1/10 or 10, several parameter pairs induced a tripartite attractor structure (electronic supplementary material, figure S4). Two sets of parameters often appeared together in these influences. First, ca_1_, cd_2_ and *γ*_4_ (dimer formation, unbinding and degradation)—which we will label ‘dimer parameters’ for shorthand. Second, *λ*_2_ and *λ*_3_ (transcription and translation)—which we will label ‘expression parameters’. Simultaneous reductions among the dimer parameters; increased *γ*_3_ with a reduced dimer parameter; and increased expression parameters with decreased cd_2_ or increased *γ*_3_ all supported the emergence of distinct attractor basins. Generally then, slower dimer dynamics and increased gene expression rates—suggestive of an influence of ATP as in [34,41–43]—support increased decision-making capacity.

Although a fully general investigation of different regulatory model structures is outside the scope of this study (see §4), we considered an alternative model structure where dimerization occurs when a monomer is bound to DNA (electronic supplementary material, table S1). Here a similar behaviour with degradation rate was observed, with distinct attractor basins emerging at higher values of γ_3_ (electronic supplementary material, figure S5). The equivalent behaviour for lower dissociation rates was not observed, likely because dimerization did not require the unbound monomeric state. We proceeded with the original model, bearing in mind that the degradation behaviour is perhaps the more general across model structures.

### 3.2. Effect of required oligomerization state of regulatory factor

The default structure of the model has the dimer of the protein product as the active regulatory factor. Following previous work exploring the effect of different degrees of model cooperativity on decision-making capacity [43], we next asked how the required oligomerization state of the regulatory protein product influenced decision-making capacity. Electronic supplementary material, figure S6, shows the more general results where gene expression is regulated by monomer, trimer and tetramer protein products. When the monomer is the active factor, most parameterizations lead to a more trivial attractor basin structure. This mirrors previous work [43] where lower Hill coefficients in a coarse-grained model led to decreased decision-making capacity—a result of the more linear, less switch-like regulatory interactions supported in the absence of cooperativity.

More unexpectedly, increasing the required oligomerization state of the regulatory factor to trimeric or tetrameric respectively showed little change and a negative change in decision-making capacity (electronic supplementary material, figure S6). The required oligomerization state of the transcription factor has the same quantitative influence on the microscopic system as an increased Hill coefficient has on the coarse-grained system—broadly increasing the nonlinearity of the increasingly step-like response [57]. In coarse-grained modelling, increasing Hill coefficients supported more attractor diversity [43]. In this microscopic case, there is a limit of oligomerization beyond which the increase in decision-making capacity stops and then reverses.

### 3.3. Influence of protein degradation and dimer–DNA dissociation on decision-making ability in the deterministic model

**Figure 3.**
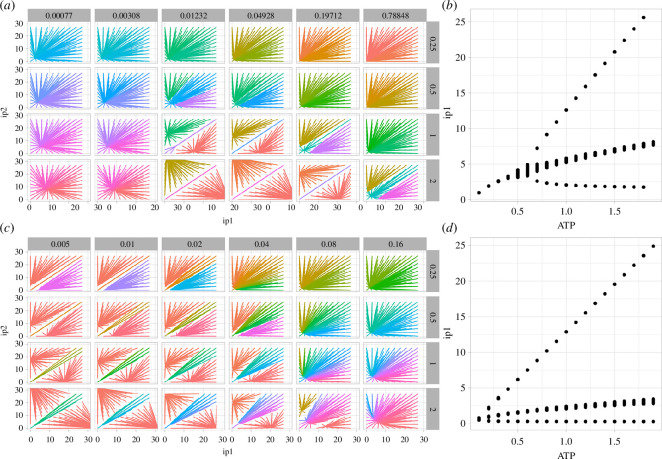
Attractor basin structure with degradation and dissociation rates, and model ATP variability. Attractor structure with (*a*,*b*) varying protein degradation rate *γ*_3_ (columns) and ATP (rows); (*c*,*d*) varying TF unbinding rate cd_2_ (columns) and ATP (rows). In (*a*,*c*), as in figure 1, each line describes how the given initial condition (ip_1_, ip_2_) evolves to a long-term position in the (p_1_*,* p_2_) space of protein expression. Colours are assigned based on the coordinates of the long-term state. In (*b*) (*γ*_3_ = 0.0077) and (*d*) (cd_2_ = 0.02), the long-term values of p_1_ are plotted as ATP increases, demonstrating the pitchfork nature of the bifurcations.

Following this exploration, we first investigated the effect of increasing the protein degradation rate *γ*_3_ in the model. The attractor basin structure in this case shows re-entrant behaviour, with a single attractor appearing for low and high degradation values, and the tripartite structure appearing for intermediate values (figure 3*a*).

We next decrease the DNA–protein dissociation rate cd_2_, so that the dimeric transcription factors stay bound to their opposing promoter regions for longer. This increased ‘memory’ induces the separation of the attractors of the system. Decreasing cd_2_ reduces the size of the central attractor so that the stability of the non-zero attractor relative to the central attractor increases as the value of cd_2_ rises.

### 3.4. ATP modulation of transcription and translation controls decision-making ability in the deterministic model

Having characterized the behaviour of decision-making capacity with some key parameters of the model, we next addressed our central question—how energy levels influence decision-making capacity. To this end, we modulate transcription and translation rates *λ*_₂_ and *λ*_3_ in the model by a factor proportional to ATP concentration, and allow this concentration to vary. The true relationship between ATP concentration and these rates has been modelled as sigmoidal [34,43] but we effectively employ a linear approximation to the centre of this sigmoidal behaviour for simplicity here.

We found that increasing model ATP concentration generally enhanced decision-making capacity in model cells, reflected in the appearance of more attractor basins through pitchfork bifurcations, and increased separation between them (figure 3). Higher ATP concentrations—and hence higher rates of gene expression—typically led to increased separation of the cell states where one protein was present at a high level and the other at a low level. In tandem, the width of the central attractor basin corresponding to balance protein levels also increased. This behaviour mirrors that observed in coarse-grained empirical models [41,43], confirming that this behaviour is not an artefact of the Hill function modelling approach and is expected to arise from the dynamics of individual regulatory interactions.

### 3.5. Alternative initial conditions and modes of ATP influence

We next considered the influence of initial differences in the other state variables of the system, and alternative models for ATP influence decoupling the effect on transcription, translation and degradation behaviours in the model. Initial differences in RNA levels had, intuitively, biasing effects on the system’s behaviour, ‘priming’ the system to favour the attractor basin corresponding to high levels of the protein with more RNA (electronic supplementary material, figure S7). Symmetrically increased RNA levels had the effect of ‘buffering’ subsequent differences in the regulatory dynamics. Here, for parameterizations supporting distinct attractors for lower initial RNA levels, higher initial RNA levels retained the single attractor structure, similar to the effects observed in [41].

To explore the fact that ATP might differently influence the different processes of gene expression, and may also have substantial influence over protein degradation, we also considered cases where ATP influence dominated transcription alone, translation alone, or transcription, translation and degradation together (electronic supplementary material, figure S8). Attractor behaviour under these alternatives was rather similar. The influence on translation alone or transcription alone was practically indistinguishable (electronic supplementary material, figure S8*a*,*b*). Including influence on protein degradation marginally expanded the range of parameters supporting distinct attractor basins, particularly at higher ATP levels, through the trend of the result that increasing ATP supports more distinct attractor basins remained robust everywhere (electronic supplementary material, figure S8*c*).

### 3.6. Stochastic modelling of cell behaviour

The above deterministic modelling suggested that increasing cellular ATP levels supports more distinct cell states, corresponding to increased decision-making capacity. We next asked whether this behaviour was supported by a yet more detailed modelling approach [25,40]—a discrete stochastic model considering the copy numbers and interactions of each individual molecule in the system. To this end, we used Gillespie’s stochastic simulation algorithm [53] to simulate trajectories of the system under different parameterizations corresponding to different cellular ATP levels.

In a stochastic model, random fluctuations can have several important influences on a system. Because random fluctuations in copy number can occur, the system does not remain forever in one attractor. Instead, fluctuations can drive the system out of the basin of one attractor and into another—corresponding to a ‘switch’ from, for example, a high p_1_, low p_2_ state into a low p_1_, high p_2_ state. The structure of attractors itself can also be different in stochastic and deterministic representations of the same system, with the same parameters. Such differences can be manifest through noise-induced bistability, where intrinsic noise can induce multistability in a system where a deterministic picture predicts only a single stable state [23–25].

**Figure 4.**
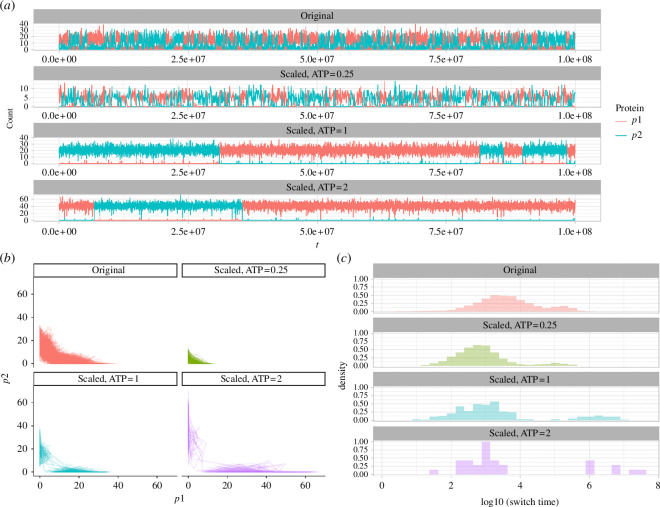
Stochastic dynamics of the model system. (*a*) Representative trajectories of protein expression levels (p_1_, p_2_) under different ATP conditions. (*b*) Trajectories of the system in (p_1_, p_2_) space over time. (*c*) Distribution of (log) switching times (the time taken for the dominant protein in the system to change). Experimental labels: Original—original parameterization from [47], with TF unbinding rate cd_2_ = 1.04. Scaled—adapted parameterization with cd_2_ = 0.04 and model ATP levels as given.

Typical stochastic trajectories of the system are shown in figure 4*a,b*. For the parameterizations considered here, switching between states corresponding to high p_1_ and states corresponding to high p_2_ is observed—with a frequency that decreases as the separation of the distinct states increases. This separation between states increases as model ATP (scaling expression rates) increases, mirroring the deterministic picture for this parameterization (though see below). Figure 4*c* shows this behaviour more explicitly—that is, for higher ATP levels, the distribution of switching times between distinct basins has more density at longer times (with some remaining density at short times due to transient behaviour around switches), corresponding to more distinct and stable cellular phenotypes, and hence more robust decision-making capacity. Taken together, the stochastic and deterministic models of this system therefore agree (internally and with existing work) that increasing ATP supply supports more, and more stable, capacity for cellular decision-making through GRN dynamics.

**Figure 5.**
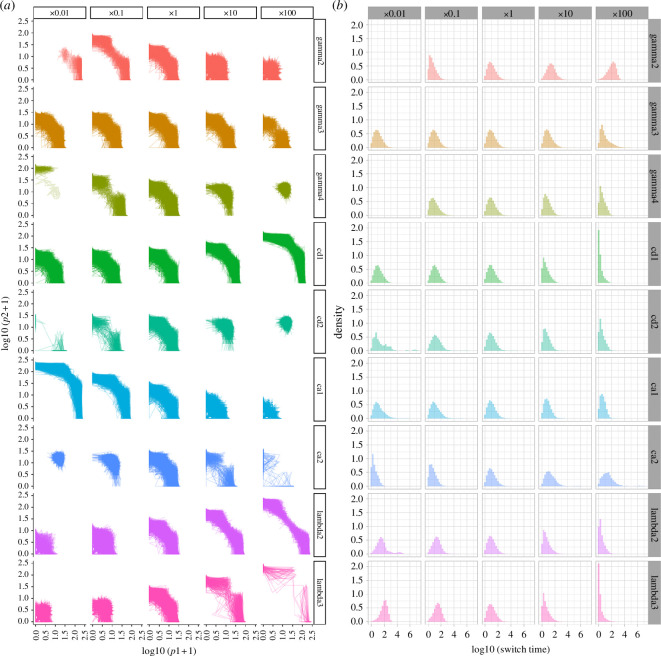
Behaviour of the system under stochastic simulation and parameter sets explored in figure 2. (*a*) Trajectories of (p_1_, p_2_) in stochastic simulation of the model where a parameter (row) is scaled by a factor (column). (*b*) Distributions of switching times between higher p_1_ and higher p_2_ states in these simulations.

To explore in more depth how the stochastic system behaved relative to the deterministic picture, we simulated stochastic behaviour under each of the parameter regimes in figure 2. The results—including trajectories of the stochastic systems, and distributions of switching times between states favouring the two proteins—are shown in figure 5.

Not unexpectedly, there are both similarities and differences between the behaviour suggested by the ODE model and that observed in stochastic simulation for the same parameter sets [23–25]. A first observation is that in those instances where the deterministic model supports distinct attractor basins, this behaviour is also observed in stochastic simulation (for example, high dimer binding ca_2_, high degradation *γ*_3_, low dimer unbinding cd_2_). These cases correspond to the most pronounced instances of distinct basins in the stochastic model, with trajectories dwelling for long periods in asymmetric states, and long tails of switching-time distributions corresponding to rare transitions between these states.

However, the stochastic model also demonstrates pronounced occupancy of asymmetric states in several instances where the deterministic model supports only a single symmetric state. Most examples agree qualitatively with the observations from pairwise changes applied to the parameters of the deterministic model (electronic supplementary material, figure S4). There, simultaneously increased ‘expression parameters’ *λ*_2_ and *λ*_3_ and decreased ‘dimer parameters’ ca_1_, cd_2_ and *γ*_4_ supported distinct attractors. In the stochastic model, individual increases in expression parameters (mirroring the modelled influence of increased ATP throughout) and decreases in the dimer parameters also lead to the confinement of the system in asymmetric states. There is then a suggestion of a parallel between the two modelling paradigms, to some extent and in this instance: those parameters that promote distinct states in the stochastic model also have a (weaker) influence in the deterministic model, whereas such promotion is manifested when coupled with a commensurate change in another parameter. But overall, the general picture that ATP**—**through influence on expression rates**—**tends to support more distinct cell states remains robust across all the modelling pictures we consider here.

## 4. Discussion

We have shown that fine-grained mechanistic models, both ODE-based and stochastic, for GRN-based cell decision-making exhibit strong dependence on ATP supply, supporting results from more coarse-grained modelling [41–43] and from different specific systems [39]. According to [43], increasing intracellular energy levels can increase the number of supported stable phenotypes, hence enhancing the capacity for decision-making. We find qualitatively similar behaviour here, with quantitative details dependent on the polymerization structure of the transcription factor governing regulation within the GRN and whether polymerization takes place on or off the DNA.

Classical studies on GRNs supporting various switch-like behaviours [44,45] consistently observe that increasing rates of gene expression stabilize and separate the attractor basins of a model. This picture aligns with our findings and those in [41,43], where the effect of increasing ATP supply is captured by increasing such rates of expression. The classic [44] also observes dependence on the steepness of the regulatory interaction between genetic components**—**specifically, that higher Hill coefficients (steeper switching) expand and stabilize an intermediate attractor basin where both genes are expressed. This expanded attractor structure agrees with the theory using coarse-grained Hill functions [43]. However, the fine-grained modelling we use here suggests some nuance to this picture**—**specifically that steeper switching (captured here by increasing the required polymerization of the regulatory factor) only stabilizes expanded attractors up to a point and that further increases limited decision-making capacity.

Our modelling approach differs from the original instance in [47] (following [44]) by the removal of the delay term in the translation process. We made this change for simplicity of interpretation and to facilitate comparison with existing modelling approaches. One consequence of this change is that the parameterization from the original paper does not demonstrate dramatic bistability in our model; changes to parameters (most directly, either through protein degradation or through TF unbinding) are needed to separate attractor basins in the system’s behavioural profile. Our rationale for focusing on a single, well-studied model for GRN-based decision-making was to provide a ‘controlled’ point of comparison between ATP dependence in a coarse-grained model [41,43] and a finer-grained model (here) for the same system. Our claim is not (cannot be) that this is the correct model for the biological processes involved but rather that the focal observation**—**ATP differences shape decision-making capacity in this model**—**does not depend on abstractions involved in previous coarser-grained representations of these. However, in parallel, we hope that the expansion of our model to different reaction structures and different parameterizations has helped support the idea that this result does not depend on a specific set of modelling choices. The investigation of ATP dependence in more general GRN structures and models, as well as parameter combinations and initial conditions outside those we have studied here, is a fascinating question which we are pursuing, but which will constitute further studies in addition to this one.

The fundamental importance of ATP of course means that it influences other cellular processes than gene expression. Cells and organisms respond to changes in ATP supply in a wide variety of ways. In humans, for example, ATP depletion can adversely affect essential cellular processes, potentially leading to organ failure and cellular dysfunction [58]. Numerous disorders have been scientifically linked to the occurrence of cellular ATP shortages. Some of these disorders include mitochondrial diseases [58], metabolic disorders, neurodegenerative diseases [59], and myopathies. In bacteria, a decline in intracellular ATP production contributes to the formation of persister cells in different species [30–32]**—**itself a decision governed by changing gene expression profiles. The general finding that limited ATP supply compromises cellular decision-making through its influence on gene expression must be interpreted in parallel with these facts that ATP responses themselves can induce cellular decisions, and the link between these ATP response decisions and the cell’s energetic ability to make them will be a target for further research.

## Data Availability

All code to reproduce the model, analysis, and visualizations is freely available at Zenodo [60]. Supplementary material is available online [61].
